# Molecular Characterization of a Recombinant Manganese Superoxide Dismutase from *Lactococcus lactis* M4

**DOI:** 10.1155/2014/469298

**Published:** 2014-01-27

**Authors:** Boon Hooi Tan, Thean Chor Leow, Hooi Ling Foo, Raha Abdul Rahim

**Affiliations:** ^1^Department of Cell and Molecular Biology, Faculty of Biotechnology and Biomolecular Sciences, Universiti Putra Malaysia (UPM), 43400 Serdang, Selangor, Malaysia; ^2^Institute of Biosciences, Universiti Putra Malaysia (UPM), 43400 Serdang, Selangor, Malaysia; ^3^Department of Bioprocess Technology, Faculty of Biotechnology and Biomolecular Sciences, Universiti Putra Malaysia (UPM), 43400 Serdang, Selangor, Malaysia

## Abstract

A superoxide dismutase (SOD) gene of *Lactococcus lactis* M4 was cloned and expressed in a prokaryotic system. Sequence analysis revealed an open reading frame of 621 bp which codes for 206 amino acid residues. Expression of *sodA* under T7 promoter exhibited a specific activity of 4967 U/mg when induced with 1 mM of isopropyl-**β**-D-thiogalactopyranoside. The recombinant SOD was purified to homogeneity by immobilised metal affinity chromatography and Superose 12 gel filtration chromatography. Sodium dodecyl sulfate-polyacrylamide gel electrophoresis and western blot analyses of the recombinant SOD detected a molecular mass of approximately 27 kDa. However, the SOD was in dimer form as revealed by gel filtration chromatography. The purified recombinant enzyme had a pI of 4.5 and exhibited maximal activity at 25°C and pH 7.2. It was stable up to 45°C. The insensitivity of this lactococcal SOD to cyanide and hydrogen peroxide established that it was a MnSOD. Although it has 98% homology to SOD of *L. lactis* IL1403, this is the first elucidated structure of lactococcal SOD revealing active sites containing the catalytic manganese coordinated by four ligands (H-27, H-82, D-168, and H-172).

## 1. Introduction

Lactic acid bacteria (LAB) are generally regarded as safe (GRAS) because they contain peptides that are readily digested in the human intestines. Due to the ability of LAB to produce large amount of lactic acid and growth inhibitory substances, they are widely used in the production of fermented food including dairy products, meat, and vegetables [1]. LAB are also vital for the production of wine, coffee, silage, cocoa, sourdough, and numerous indigenous food fermentations [2]. The importance of LAB in human health is becoming more significant since they are GRAS microbe and natural. Apart from being manufactured as probiotics, LAB could also be used as vehicles for the delivery of pharmaceutical or nutraceutical agents [3].

Among the LAB that are widely used for the production of fermented food products is *Lactococcus lactis*. However, exposure of *L. lactis* to various environmental stresses during industrial processes has triggered deleterious effect to the cells, such as oxidative toxicity that can cause cellular damage at both molecular and metabolic levels [1]. In order to deal with oxidative stress, *L. lactis* is equipped with general and specific stress response mechanism, one of which is accomplished by the activity of superoxide dismutase (SOD).

SOD plays a vital role in the defense mechanism against the oxidative stress which is caused by reactive oxygen species (ROS), such as superoxide radicals (O_2_^−^), hydrogen peroxide (H_2_O_2_), and hydroxyl radical (^•^OH). These ROS impose oxidative damage to the cells, including DNA strand breakage, protein inactivation, and membrane lipid peroxidation [4]. SOD protects living organism from oxidative damage by catalyzing the formation of H_2_O_2_ and O_2_ from O_2_^−^ [5].

SOD can be classified into four groups according to their metal cofactor: manganese (MnSOD), iron (FeSOD), copper-zinc (CuZnSOD), and nickel (NiSOD). MnSOD, encoded by *sodA*, is found in prokaryotes and in mitochondria matrix of eukaryotes [6]. MnSOD and FeSOD are structurally very similar, whereas CuZnSOD is not related [7]. SOD can be found in almost all aerobic and some anaerobic organisms. All previously tested streptococci (including *Lactococcus lactis* subsp. *lactis*) appear to carry a MnSOD [8].

Previous study has discovered a unique manganese-containing SOD in *L. lactis* during an analysis of acid stress-induced protein expression [9]. However, the shortcoming of this *sodA* is that it has a low initial expression. Sufficient amount of SOD is necessary for the characterization study. This problem was solved with recombinant DNA techniques that facilitate analysis of the gene and for long term storage, as well as obtaining substantial protein in a shorter period. In this study, a full-length SOD gene from a locally isolated *L. lactis* M4 was cloned into pRSET-A expression vector that utilizes the T7 promoter system and was expressed in *E. coli* BL21(DE3)pLysS for inducible high-level protein expression. Purification and characterization of the *L. lactis* SOD was carried out in order to provide a better understanding of its physiological and biochemical aspects which may serve as a basis to improve the survival of lactococcal cells. The first predicted structure for lactococcal MnSOD was also elucidated.

## 2. Materials and Methods

### 2.1. Bacterial Strains, Plasmids, and Growth Conditions

*L. lactis* M4 was a locally isolated strain from fresh milk. *E. coli* BL21(DE3)pLysS, *E. coli* TOP10, pCR-BluntII-TOPO vector, and pRSET A expression vector were purchased from Invitrogen (Invitrogen, USA). *L. lactis *M4 was grown at 30°C in M17 medium supplemented with 0.5% glucose. *E. coli* BL21(DE3)pLysS harboring and *E. coli* TOP10 harboring pCR/SOD were cultured aerobically at 37°C in Luria Bertani (LB) medium supplemented with 35 *μ*g/mL chloramphenicol and 50 *μ*g/mL ampicillin or 10 *μ*g/mL kanamycin, respectively.

### 2.2. Cloning of SOD Gene

Total genomic DNA was extracted from* L. lactis *M4 by the methods developed by Engelke et al. [10]. Plasmid DNA was isolated using Wizard *Plus* SV Minipreps DNA Purification System (Promega, USA). Agarose gel electrophoresis was carried out to analyze total genomic DNA fragments and plasmid on 0.8% and 1% (w/v) agarose gel, respectively. The gel was then stained with ethidium bromide and observed under a 300 nm UV transilluminator.

PCR primers for the amplification of SOD gene were designed based on *L. lactis* subsp.* cremoris* MG1363 *sodA* gene sequence (GenBank accession no. U17388). The primers were sod_fwd (5′-CGC CTC GAG **ATG** GCA TTT ACA TTA CCT GAA CTT CCA TAT GC-3′; *Xho*I) and sod_rev (5′-GCG AAG CTT TTA TTT TGC CTT AGC GTA AAG TTC ATT GAC-3′; *Hin*dIII). Restriction enzyme sites for *Xho*I and *Hin*dIII were incorporated into the primers (underlined; start codon showed by bolding of the alphabets). Screening and amplification of the full-length SOD gene from *L. lactis* M4 were carried out by PCR using Mastercycler (Eppendorf, Germany). Total volume of the reaction mixture which consisted of 2.5 *μ*L 10X reaction buffer (Fermentas, Germany), 0.5 *μ*L 10 mM dNTP (Fermentas, Germany), 0.5 *μ*L 10 *μ*M forward primer, 0.5 *μ*L 10 *μ*M reverse primer (First Base, Malaysia), 1 *μ*L *Pfu* DNA polymerase 2.5 U/*μ*L (Fermentas, Germany), 6 *μ*L DNA template, and 14 *μ*L distilled water PCR was carried out with an initial denaturation step at 95°C for 5 min followed by a subsequent denaturation step for 1 min at the same temperature. It was then followed by annealing at 55°C for 1 min 30 sec and extension at 72°C for 1 min for 34 cycles. Finally, a 7 min extension step was run at 72°C.

*Xho*I and *Hin*dIII (Fermentas, Germany) were used in restriction enzymes digestion which was carried out at 37°C for 3 h. PCR product was extracted from agarose gel by using the QIAquick Gel Extraction Kits (Qiagen, Germany). The purified PCR product was first cloned into pCR-BluntII-TOPO vector and then subcloned into pRSET A expression vector. The constructed plasmids were transformed into competent *E. coli* BL21(DE3)pLysS. Ligation was carried out at 16°C for 8 h before transformation. Incubation period for transformation was set at 16 h at 37°C. LB agar plates containing 50 *μ*g/mL ampicillin and 35 *μ*g/mL chloramphenicol were used to select the transformants. Restriction enzymes digestion analysis was performed to screen the presence of the insert.

### 2.3. Sequence Analysis

The constructed pRSET/SOD was sent for automated nucleotide sequencing using ABI 3730 DNA Analyzer (Applied Biosystem, USA). Analyses of the nucleotide sequence and the deduced amino acid were done by using program BLAST (BLASTN and PSI-BLAST) from NCBI (http://www.ncbi.nlm.nih.gov) and BioEdit software (version 7.0.5.3).

### 2.4. Expression and Purification of SOD

The recombinant *E. coli* cells were cultured at 37°C in 1 L LB broth with vigorous shaking. Isopropyl-*β*-d-thiogalactopyranoside (IPTG) was added to a concentration of 1 mM to induce the expression of the recombinant protein. The culture was incubated at 37°C for 2 h with vigorous shaking. Then, the bacterial cells were harvested by centrifugation at 4000 ×g for 10 min. The cell pellet was resuspended with 30 mL of lysis buffer (0.1 M phosphate buffer, 0.5 M NaCl, pH 7.0) and vortexed for approximately 2 min. Extraction of the recombinant protein prior to purification was done by physical method using glass beads. The mixture was vortexed for 1 min and then cooled on ice for another 1 min. This procedure was repeated three times before centrifugation at 10,000 ×g for 5 min. The supernatant was collected and kept at 4°C until further use.

All liquid chromatographies was carried out using ÄKTA Purifier FPLC system (GE Healthcare, USA). The supernatant was dialyzed against 2.6 L of Buffer A (20 mM phosphate buffer, 0.5 M NaCl, 30 mM imidazole, pH 7.0) overnight at 4°C with three changes of buffer. The dialyzed sample was subjected to purification on a HisTrap HP column (GE Healthcare, USA) preequilibrated with Buffer A. The His-tagged protein that bound to the nickel ions (Ni^2+^) in the column was then eluted with Buffer B (20 mM phosphate buffer, 0.5 M NaCl, 300 mM imidazole, pH 7.0) at a flow rate of 1 mL/min at room temperature. The elution profile was monitored at 280 nm. Fractions were collected and assayed for SOD activity. The fractions from immobilised metal affinity chromatography (IMAC) with SOD activity were pooled and dialyzed against Buffer C (20 mM phosphate buffer, 0.15 M NaCl, pH 7.0) overnight at 4°C with three changes of buffer. The dialyzed enzyme was then applied to a Superose 12 HR 16/70 packed column (GE Healthcare, Malaysia), which was preequilibrated with Buffer C at a flow rate of 0.75 mL/min. Aarotinin (6.5 kDa), cytochrome *c* (12.4 kDa), carbonic anhydrase (29 kDa), and bovine serum albumin (66 kDa) were used as the protein molecular weight standard marker. Absorbance at 280 nm was monitored and SOD activity was assayed for each fraction. The SOD active fractions were pooled and dialyzed against 50 mM phosphate buffer, pH 7.0. The protein concentration was measured using Bradford method [11] with bovine serum albumin (BSA) as the reference standard. The purified enzyme was stored at 4°C for subsequent studies.

### 2.5. Protein Detection and Analysis

Total protein was denatured by boiling for 5 min. Electrophoresis was carried out at 250 V for 45 min on a 12% (w/v) denaturing polyacrylamide gel to separate the protein according to the protocol of Laemmli [12]. Sodium dodecyl sulfate-polyacrylamide gel electrophoresis gel (SDS-PAGE) consisted of 12% resolving gel [4 mL of 30% (w/v) acrylamide/bis solution, 2.5 mL of resolving buffer, 0.1 mL of 10% SDS, 3.35 mL of dH_2_O, 10 *μ*L of TEMED, and 60 *μ*L of freshly prepared 10% (w/v) ammonium persulphate] and 4% stacking gel [2.3 mL of 30% (w/v) acrylamide/bis solution, 2.5 mL of stacking buffer (pH 6.8), 0.1 mL of 10% (w/v) SDS, 10 *μ*L of TEMED, 60 *μ*L of freshly prepared 10% (w/v) ammonium persulphate, and 5.1 mL of dH_2_O]. For protein analysis, 10 *μ*L of samples was loaded into each well. Coomassie Brilliant Blue staining solution was used to stain the electrophored proteins. The separated recombinant protein of the polyacrylamide gel was transferred to polyvinylidene difluoride (PVDF) membrane for western blot analysis. Immunoblotting was carried out using western MAX HRP kit (Amresco, USA). The membrane was blocked with DBTB [Dilution Buffer Powder (1.2 g in 100 mL dH_2_O) with 1% (v/v) Tween 20 (DBT), plus 1% (w/v) BSA] for 30 min at room temperature with agitation. Monoclonal anti-His antibody (anti-HisG antibody) against the fused N-terminal 6xHis-tag was used to detect the presence of the recombinant protein. The membrane was then incubated with primary antibody (monoclonal anti-His antibody) at room temperature for 30 min. The primary antibody was aspirated and the blot was washed with an ample amount of DBT for 5 min with agitation. Then, the DBT was aspirated. The washing step was repeated twice. The horseradish peroxidase (HRP) conjugated goat anti-IgG secondary antibody bound to the monoclonal anti-His antibody was added until enough to cover the membrane. Then, the membrane was incubated for 30 min with agitation. The secondary antibody was then discarded and the membrane was washed with DBT for 3 times. DAB (3,3′-diaminobenzidine) substrate (10 mL) was used to detect the secondary antibody at room temperature until brown color developed.

SOD activity on native PAGE was monitored by nitrobluetetrazolium (NBT) negative staining system [13, 14]. Purified enzyme was electrophoresed on a 10% (w/v) native polyacrylamide gel at 100 V for 1 h. After that, the gel was soaked in 25 mL of 1.23 mM NBT for 15 min, briefly washed, and then soaked in 30 mL of 100 mM phosphate buffer (pH 7.0) containing 28 mM TEMED and 28 *μ*M riboflavin for another 15 min in the dark. The gel was briefly washed again before exposing to light for 15–30 min. The activity of SOD was revealed as the achromatic bands on the gel.

### 2.6. Enzyme Characterization

Isoelectric focusing (IEF) was performed on 1 mM thick Ampholine PAG plate, pH 3.5–9.5 (GE Healthcare, USA) by using Multiphor II system according to the manufacturer's protocol. The pI value of each band visualized on the gel was estimated based on the Broad range pI marker, ranging from pH 3 to 10 (GE Healthcare, USA). The SOD activity of IEF-PAGE resolved proteins was determined by SOD activity staining method. Purified SOD was assayed at various temperatures (4–65°C) and pH (4.0 to 11.0) for optimum temperature and pH studies. The thermal stability of SOD was determined by incubating the purified SOD in 50 mM phosphate buffer (pH 7.8) at a series of elevated temperatures (25–60°C) for 60 min prior to assay for SOD activity. For the effect of chemicals and inhibitors, sodium azide (NaN_3_), potassium cyanide (KCN), H_2_O_2_, SDS, and ethylenediaminetetraacetic acid (EDTA) with different concentrations (0.1 mM, 1 mM, and 5 mM) were tested. The enzyme solution containing each compound was incubated in 50 mM phosphate buffer (pH 7.8) at optimum temperature for 20 min and then assayed for enzyme activity.

### 2.7. SOD Assay in Solution

SOD activity was determined after each purification step using the photochemical microplate assay method [13, 15], by measuring its ability to inhibit the photochemical reduction of nitrobluetetrazolium (NBT). The reaction mixture contained 50 mM phosphate buffer (pH 7.8), 13 mM methionine, 75 *μ*M NBT, 2 *μ*M riboflavin, 0.1 mM EDTA, and 0–12 *μ*L enzyme extract. Riboflavin was added last into the reaction mixture. The microplate was placed 30 cm below two 40-watt lamps and the reaction was run for 15 min. Absorbance was read using an ELISA reader at 560 nm. Reaction mixture without enzyme (as control) developed maximum color, whereas the intensity of the color decreased with the increasing volume of enzyme extract. One enzyme unit is equal to 50% inhibition of the reaction.

### 2.8. Structure Prediction of SOD

The templates for modeling of SOD structure were searched from PSI-BLAST database at National Center for Biotechnology Information (http://www.ncbi.nlm.nih.gov/BLAST). Homology modeling was performed using YASARA [16] with 1JR9 [17] and 2RCV [18] as templates. The predicted structures were refined using simulated annealing minimization approach in YASARA, where the final model was evaluated using Ramachandran plot, Verify 3D, and Errat programmes. Superposition of *Cα* traces of model and template structures was performed using Swiss-PdbViewer version 4.0.1 [19].

## 3. Results and Discussion

### 3.1. Cloning and Expression of the SOD Gene in *E. coli*

**Figure 1:**
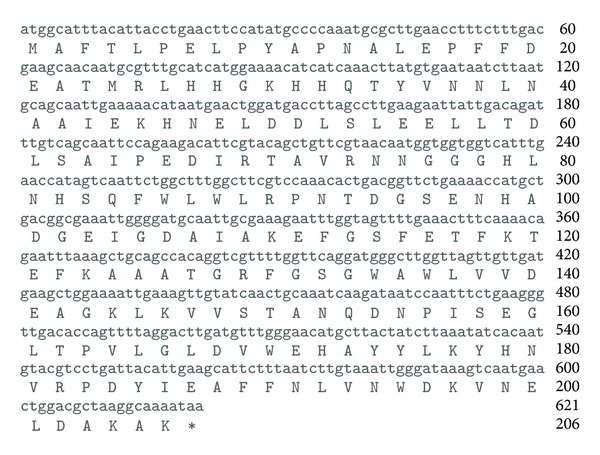
Nucleotide sequence and deduced amino acid sequence of SOD gene from pRSET/SOD. The asterisk denotes the stop codon.

**Figure 2:**
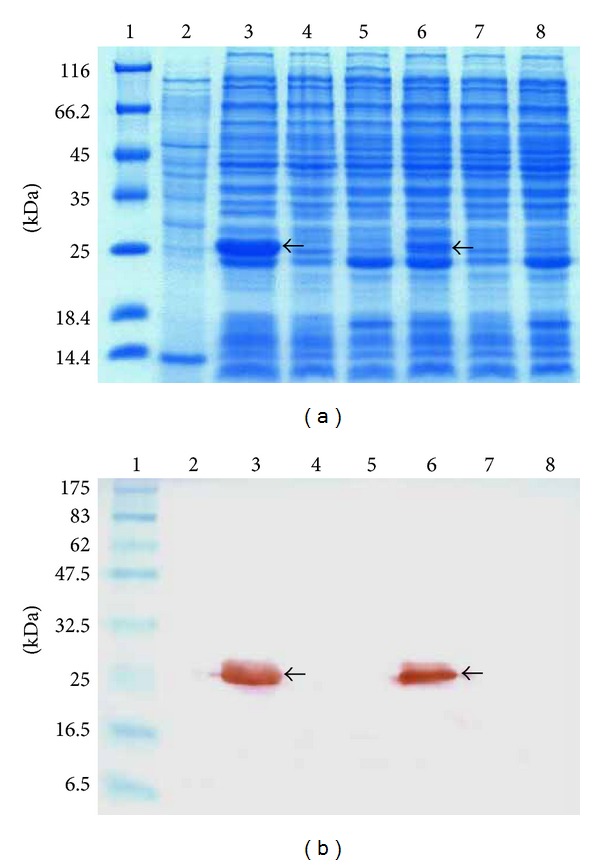
Analysis of denatured protein on 12% SDS-PAGE (a) and detection of recombinant SOD on western blotting PVDF membrane (b). Lane 1(a): protein molecular weight marker; lane 1(b): prestained protein marker (NEB); lane 2: crude extract of *L. lactis* M4; lane 3: pRSET/SOD; lane 4: pRSET A; lane 5: *E. coli* BL21(DE3)pLysS were with the addition of 1 mm IPTG; lane 6: pRSET/SOD; lane 7: pRSET A; lane 8: *E. coli* BL21(DE3)pLysS were without the addition of 1 mM IPTG. Arrows indicate the presence of the expressed SOD at about 27 kDa after staining using Coomassie Brilliant Blue staining for SDS-PAGE and detecting of horseradish peroxidase (HRP) conjugated secondary antibody with 3,3′-diaminobenzidine (DAB). The intensity of the recombinant SOD protein band with IPTG induction is significantly higher than the noninduced recombinant SOD.

A gene encoding superoxide dismutase (SOD) was amplified from *L. lactis* M4 on the basis of *L. lactis* subsp. *cremoris* MG1363 *sodA* sequence. The gene was cloned into pCR-Blunt II-TOPO and then subcloned into pRSETA expression vector under the regulation of T7 promoter. Transformants containing the constructed plasmid (pRSET/SOD) were selected on LB agar plates containing appropriate antibiotic. Positive clones were verified by PCR, restriction enzyme digestion analysis, and sequencing. BLASTN analysis of the sequencing result showed 98% identity to the published nucleotide sequence *sodA* in the genome of *L. lactis *subsp. *lactis *IL1403 (accession number AE005176) and 99.5% identity to respective SOD sequence of *L. lactis* subsp. *lactis* IL1403. These results showed that the full-length *sodA* from *L. lactis* M4 (accession number FJ905108) comprised 621 nucleotides that could encode a protein of 206 amino acids (Figure 1). The only variant of amino acid was found at amino acid 202 of SOD, where it was Tyr and Asp for *L. lactis* subsp. *lactis* IL1403 and *L. lactis* M4, respectively. Expression of the recombinant SOD in *E. coli* BL21(DE3)pLysS was induced with 1 mM of IPTG. SDS-PAGE of the total expressed protein showed an overexpressed protein band of about 27 kDa in molecular mass (Figure 2(a)), which is in agreement with the combined molecular mass of lactococcal SOD (24 kDa) and 6xHis-tag (3 kDa).

Western blot analysis confirmed the expression of the recombinant SOD by the fusion of monoclonal anti-His antibody (Anti-HisG antibody) against the N-terminal 6xHis-tag located upstream of *sodA* gene (Figure 2(b)). Both of the induced and uninduced SOD were detected by western blot, but the intensity of the induced SOD was higher than the uninduced SOD.

### 3.2. Purification and Characterization of SOD

**Figure 3:**
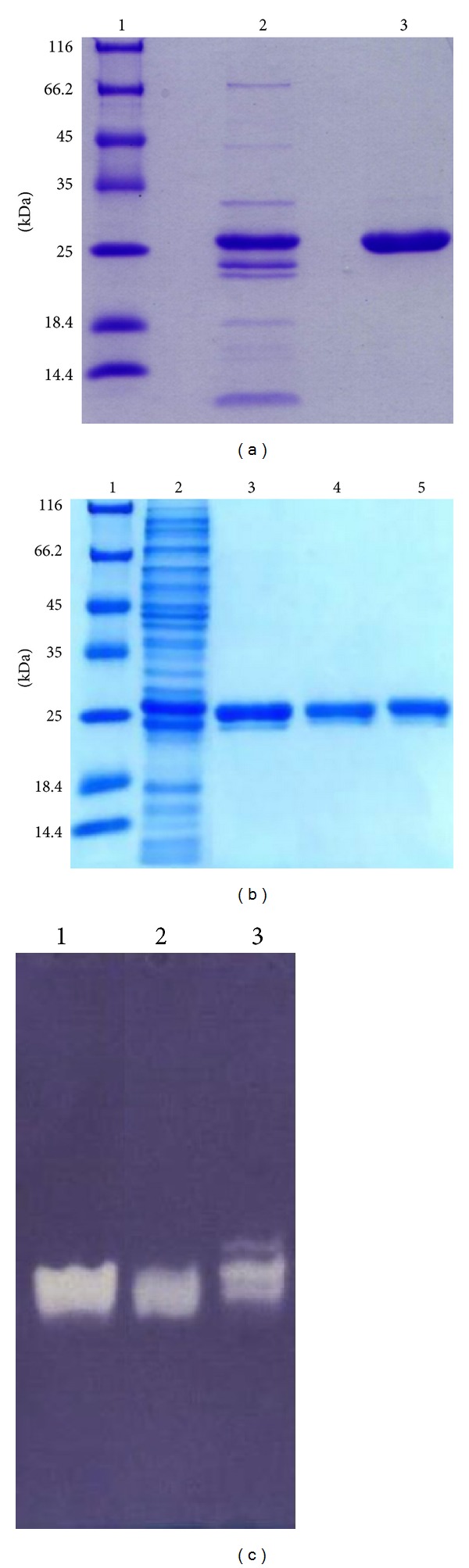
SDS-PAGE analysis of the pooled fractions after each purification step. SDS-PAGE was performed on 12% denatured polyacrylamide gel. (a) SDS-PAGE analysis of pooled fractions with SOD activity after IMAC: M, protein molecular weight marker (Fermentas); lane 1, pooled peak 1 (6 *μ*g); lane 2, pooled peak 2 (6 *μ*g); (b) SDS-PAGE analysis of pooled fractions after gel filtration: M, protein molecular weight marker (Fermentas); lane 1, crude extract (10 *μ*g); lane 2: pooled SOD active fractions (peak 2) after IMAC (4 *μ*g); lane 3: pooled SOD active fractions after gel filtration chromatography (2 *μ*g); lane 4: dialyzed pooled SOD active fractions after gel filtration chromatography (2 *μ*g); (c) SOD activity staining of the purified native SOD electrophoresed on 10% nondenatured polyacrylamide gel. Lane 1: IMAC; (2) gel filtration; (3) dialyzed sample. The achromatic zone against the purple background revealed the activity of SOD. Each lane is loaded with 2 *μ*g of protein.

The purification procedure of recombinant SOD is summarized in Table 1. The purity and the estimated molecular weight of recombinant SOD were analyzed with SDS-PAGE after each purification step (Figure 3). The dialyzed recombinant SOD was purified to apparent homogeneity by IMAC and gel filtration chromatography having a purification fold and yield of 3.74 and 22.84%, respectively. The specific activity of purified enzyme was 1.865 × 10^4^ units/mg protein. SOD activity of purified enzyme was visualized on native polyacrylamide gel (PAG) by NBT activity staining. The negatively stained bands against the purple color background of the PAG indicate the activity of SOD (Figure 3(c)).

**Figure 4:**
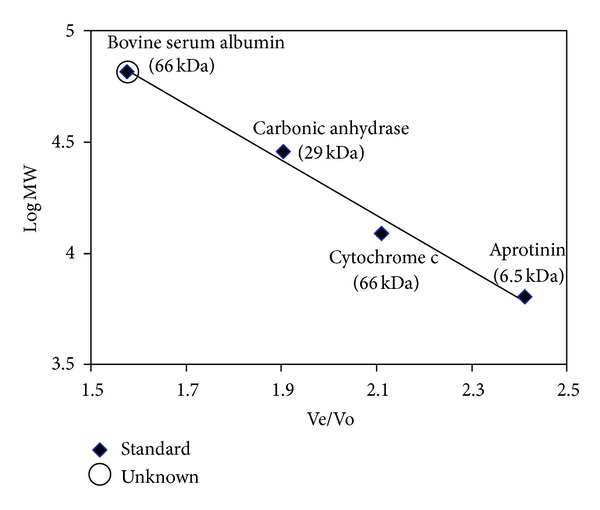
Determination of molecular weight by size exclusion chromatography. Chromatography was performed on Superose 12 HR 17/70 packed column at a flow rate of 0.75 mL/min with PBS (20 mM phosphate buffer, 0.15 M NaCl, pH7).

Size exclusion chromatography revealed that the dimer elutes at lower Ve/Vo with estimated molecular mass of 63 kDa (Figure 4), slightly larger than the theoretical molecular weight (MW) of 55.4 kDa for dimer (MW monomer ~27.7 kDa). The theoretical molecular weight was in agreement with the deduced denatured SOD subunit of 27 kDa by SDS-PAGE. The discrepancy in size could be due to a less compact organization as the 39 amino acid residues coded by vector arm were attached at N-terminus of SOD. This finding postulated a dimeric structure of the recombinant SOD that was in agreement with the commonly reported dimeric prokaryotic MnSOD. All known MnSODs are either homodimers or homotetramers with subunit molecular weights of about 20 kDa. Most of the eukaryotic MnSODs are tetrameric, including human MnSOD [20]. The prokaryotic MnSODs are usually dimeric, except for the extreme thermophiles *Thermusthermophilus* and *Thermusaquaticus* which have tetrameric MnSODs [21, 22]. *E. coli* MnSOD is a homodimer with subunit molecular mass of 21.6 kDa [23].

**Figure 5:**
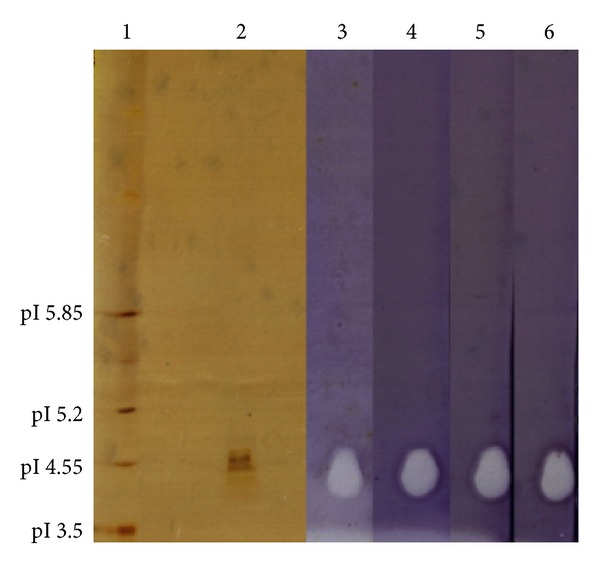
IEF PAGE analysis and SOD activity staining of purified SOD. Each lane contained 1 *μ*g of purified SOD. Lane 1: broad range pI marker; lane 2: purified SOD with pI value of 4.5; lane 3: purified SOD without any treatment as control; lane 4: SOD treated with 2 mM KCN; lane 5: SOD treated with 3 mM H_2_O_2_; and lane 6: SOD treated with 2 mM KCN and 3 mM H_2_O_2_. The achromatic zones of the control and the treated enzyme showed no significant differences.

Isoelectric focusing analysis on the purified SOD has shown a pI of 4.5, which is closely related to the calculated pI of *L. lactis* sp. *cremoris* MG1363 [9]. When tested with the inhibitors, the results showed that there was no significant difference between the achromatic zones in activity stained gel of the enzyme before and after treatment with cyanide and H_2_O_2_, indicating the activity of this enzyme was not inhibited by cyanide or H_2_O_2_ (Figure 5). The isoforms of SOD can be distinguished by their different sensitivities to cyanide and H_2_O_2_. FeSOD is irreversibly inactivated by H_2_O_2_ [24], while CuZnSOD is inhibited by cyanide [25]. MnSOD is resistant to both cyanide and H_2_O_2_ [15, 24, 25]. Therefore, the insensitivity of this lactococcal SOD to these two inhibitors confirmed that it was a manganese SOD (MnSOD).

**Figure 6:**
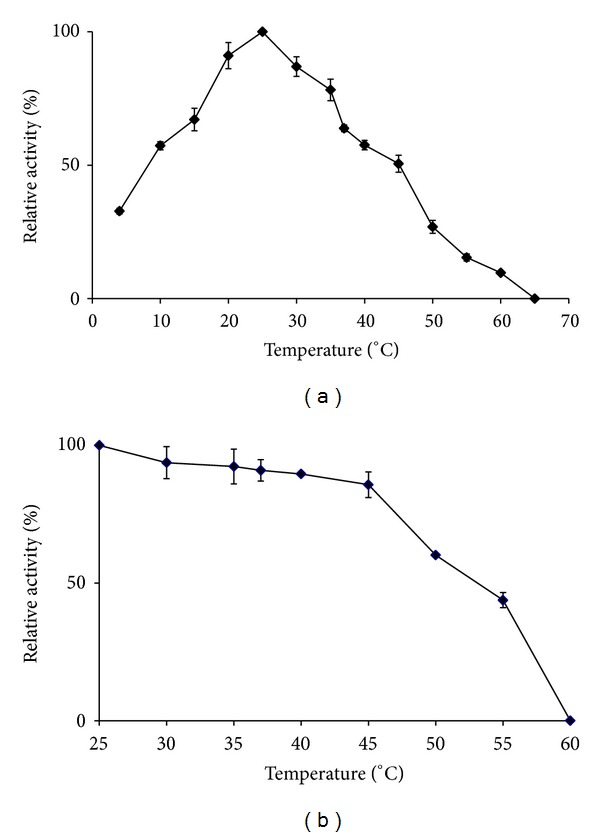
Effect of temperature on SOD activity (a) and stability (b). The purified SOD was assayed at different temperatures (5 to 65°C). Thermostability of SOD was measured by incubating SOD at various temperatures (25–60°C) for 60 min prior to SOD assay at its optimum temperature.

The SOD was highly active between 20°C and 30°C with an optimum temperature at 25°C. The enzyme was thermostable up to 45°C by retaining more than 85% of SOD activity. Further treatment above 45°C caused the activity to decrease drastically and completely deactivated at 60°C (Figure 6), indicating it was susceptible to thermal inactivation. These observations suggest that SOD may not be involved in the heat shock or cold shock regulation in *L. lactis*. Lactococcal cells grow at low temperatures by merely slowing down biological processes whereas growth at high temperature is deleterious to the cell [26]. In support of the results obtained in this work, most of the MnSODs are stable in the range from 25 to 45°C, except for MnSODs derived from the thermophiles which exhibited higher stability [27] due to thermal adaptation at functional environment.

Optimum SOD activity was obtained at pH 7.2. The enzyme retained more than 70% of its maximum activity between pH 7 and 8. However, the enzyme lost its activity substantially under alkaline conditions but still retained about 20% SOD activity at pH 11. The enzyme was also inactivated in acidic condition at pH below 6. This enzyme is susceptible to acid stress because acidic pH favors the dissociation of the functional tetramer into monomers and thus affects the enzyme activity. Study conducted by Ken et al. [28] had revealed that acidic pH favors monomer formation and suggested that the charge interaction could be important for subunit association.

Effects of various chemicals and inhibitors at different concentrations on the SOD activity showed that it was strongly inhibited by SDS and moderately inhibited by NaN_3_ and EDTA, while the inhibition by KCN and H_2_O_2_ was not significant (Table 2). Specific inhibitors for SOD have been useful in many biochemical studies particularly in distinguishing types of SOD. *L. lactis* M4 SOD was insensitive to H_2_O_2_ and KCN, confirming that it belongs to the class of MnSOD. In fact, whilst FeSOD and MnSOD are resistant to KCN, FeSOD is however irreversibly inactivated by H_2_O_2_ [27–29]. Azide (NaN_3_) inhibited SODs differently where the sensitivity depends on the metal prosthetic groups. FeSOD is more susceptible than MnSOD with CuZnSOD being the least sensitive towards inhibition by azide (NaN_3_) [29, 30]. The manganese is tightly bound to the ligands H-27, H-82, D-168, and H-172 and hardly removed by lower concentration of chelator EDTA especially at function pH above the pKa values of histidine and aspartate.

### 3.3. Homology Modeling of MnSOD

**Figure 7:**
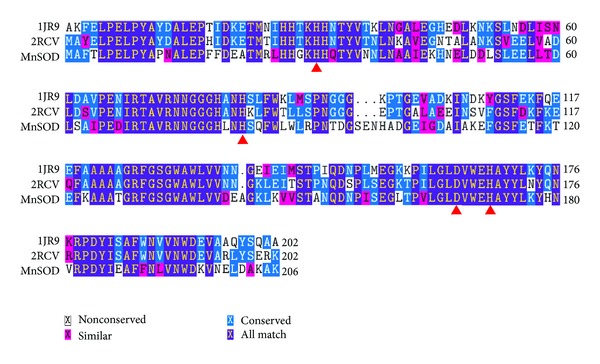
Sequence alignment of MnSOD and its template sequences. The sequence alignment was generated using CLUSTALW and TEXSHADE in Biology Workbench 3.2 (http://workbench.sdsc.edu/). The alignment was generated using SOD of *Lactococcus lactis* M4 (FJ905108), SOD (PDB accession number 1JR9) of *Bacillus halodenitrificans*, and SOD (PDB accession number 2RCV) of *Bacillus subtilis*. The binding sites of active-site metal Mn are marked with red triangles.

**Figure 8:**
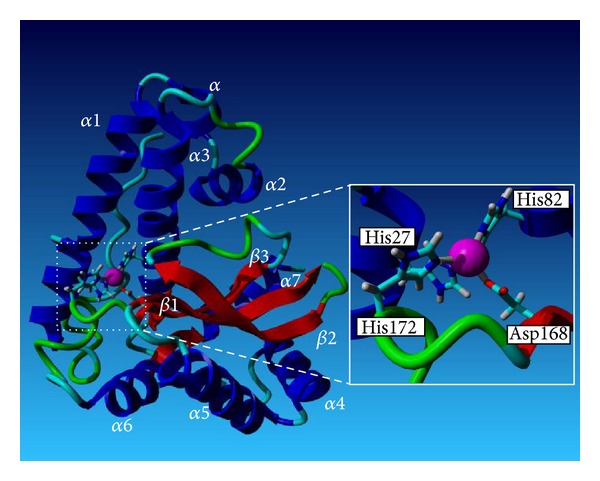
The predicted structure of MnSOD with secondary structure rendered as ribbon. The secondary structure assignments were named according to their sequence on polypeptide chain except helix *α* due to its short length. The boxed segment is the close-up view of active center.

A PSI-BLAST was performed to search for homologous crystal structure in the NCBI database. Crystal structures of *Bacillus halodenitrificans* (1JR9) and *Bacillus subtilis* (2RCV) were chosen as templates because no crystal structure of lactococcal MnSOD has yet been elucidated. Multiple sequence alignment between MnSOD and crystal structures 1JR9 and 2RCV showed a number of structurally conserved regions (SCRs) of 61.2% and 60.7%, respectively (Figure 7). The structural information of 1JR9 (2.8 Å) and 2RCV (1.6 Å) was extrapolated to MnSOD by extracting the coordinates of the protein backbone of crystal structures and modeled onto MnSOD using YASARA. Model 2 was slightly better than Model 1 as it is validated using PROCHECK [31], Verify-3D [32], and Errat [33] because Model 2 was derived from a better quality template 2RCV (1.6 Å) as compared to Model 1 which used 1JR9 (2.0 Å) as template. The root-mean-square deviation (RMSD) between the alpha carbon (C*α*) atoms of Model 1/1JR9 and Model 2/2RCV was 0.5 Å and 0.47 Å, respectively, despite being with only about 60% amino acid sequence identity. Alignment of MnSOD with crystal structures 1JR9 and 2RCV accurately annotated active-metal Mn binding sites as it was more conserved than the rest of amino acid sequences. The active site Mn is stabilized by close coordination with three histidines (His27, His82, and His 172) and one aspartic acid (Asp168) as revealed in Figure 8. The ligands for Mn are NE2 of His27, NE2 of His82, OD1 and OD2 of Asp168, and NE2 of His172 at distances of 1.878 to 2.384 Å for Model 1 and 1.906 to 3.129 Å for Model 2.

To date, four types of SODs have been reported based on the metal species at the active site: copper/zinc, nickel, manganese, and iron [34]. The predicted structure of *L. lactis* M4 SOD comprises *α*-helical domain and *α*/*β* domain. The *α*-helical domain contains two long *α*-helices, *α*1 (Asp20-Glu44) and *α*3 (Ile68-Leu89) which are connected by two short *α*-helices, namely, *α* (His46-Asp51) and *α*2 (Ser53-Asp60), whereas *α*/*β* domain comprises two *α*-helices, *α*4 (Gly102-Phe112) and *α*5 (Ser114-Gly128), followed by three-*β* strands, *β*1 (Gly133-Asp140), *β*2 (Gly143-Thr150), and *β*3 (Thr162-Asp168), and additional two *α*-helices, *α*6 (Val181-Phe190) and *α*7 (Asn194-Ala205). The major differences between M4 SOD and 1JR9 were the antiparellel *β*-sheet formed by *β*1, *β*2, and *β*3, where the *β*-strands were shorter for 1JR9 [17]. The loop between *α*3 and *α*4 for M4 SOD was slightly longer than that of 1JR9 and 2RCV. The predicted structure of *L. lactis* M4 SOD contains one manganese ion coordinated by four residues (H-27, H-82, D-168, and H-172), with two of the Mn-coordinating residues originating from the *α*-helical domain and the remaining two arising from the *α*/*β*-domain. This two-domain subunit fold is a typical feature of MnSODs [18] and thus it is postulated that *L. lactis* M4 SOD is a MnSOD.

## 4. Conclusion

MnSOD of *L. lactis* M4 was present as dimeric structure as revealed by gel filtration chromatography. The predicted *L. lactis* M4 SOD structure will provide a basis for understanding of the structure and function of lactococcal MnSOD. Further understanding on the biological function of MnSOD through X-crystallography is helpful to unveil the biological function of dimeric *L. lactis* M4 MnSOD. It is important to unlock the catalytic mechanism of *L. lactis* M4 SOD in removing oxidative damage.

## Figures and Tables

**Table 1:** Summary of the purification procedure for the SOD from *Lactococcus lactis* M4.

	Total activity (U)	Total protein (mg)	Specific activity (U/mg)	Yield (%)	Purification fold
Crude extract	16,982	88.9	4,967	100.00	1.00
IMAC	7,504	12.5	8,706	44.19	1.75
Gel filtration	3,796	7.1	18,076	22.35	3.64
Dialysis	3,879	6.9	18,650	22.84	3.75

Note: IMAC represents immobilized metal affinity chromatography. Two peaks of active SOD activity were detected after IMAC. Only peak 2 was subjected to further purification steps.

**Table 2:** Effect of chemicals and inhibitors on the activity of the purified SOD.

Inhibitors	Inhibition of SOD activity (%)		
	0.1 mM	1 mM	5 mM
H_2_O_2_	1.3	4.6	9.6
KCN	1.7	5.1	10.3
NaN_3_	18.9	34.4	48.3
EDTA	5.7	11.6	26.4
SDS	29.2	100.0	100.0
